# Square-Wave Voltammetry Enables Measurement of Light-Activated Oxidations and Reductions on n-Type Semiconductor/Metal Junction Light-Addressable Electrochemical Sensors

**DOI:** 10.1021/acs.analchem.3c00630

**Published:** 2023-06-05

**Authors:** Enock G. Arthur, Hana Ali, Armeen Hussain, Glen D. O’Neil

**Affiliations:** † Department of Chemistry and Biochemistry, Montclair State University, Montclair, New Jersey 07043, United States; ‡ Sokol Institute for Pharmaceutical Life Sciences, Montclair State University, Montclair, New Jersey 07043, United States

## Abstract

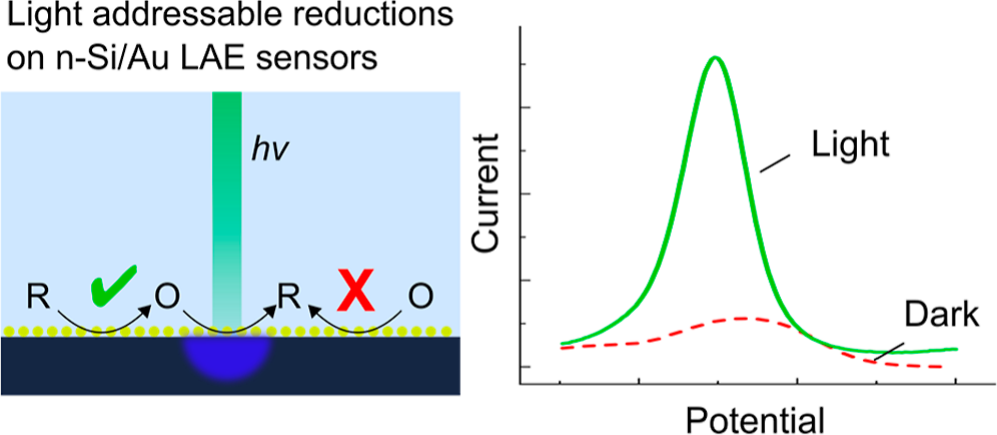

Light-addressable electrochemical (LAE) sensing is a photoelectrochemical technique that enables high-density, individually addressed electrochemical measurements using light to activate an electrochemical reaction at the surface of a semiconducting photoelectrode. However, one major challenge is that only one electrochemical reaction (oxidation or reduction) will be activated by light. Here, we used square-wave voltammetry (SWV) to enable measurement of both types of electrochemical reactions using n-Si/Au NP LAE sensors. We demonstrated this approach for the oxidation of ferrocene methanol and the reduction of ruthenium hexamine and methylene blue. We found that for all molecules, SWV showed dramatic improvements in current under illumination in comparison with dark samples. We also demonstrated that this approach works for both fully illuminated and partially illuminated samples. Altogether, we hope these results open up new applications for LAE sensors, especially those based on semiconductor/metal junctions.

## Introduction

Light-addressable electrochemical sensing (LAES) is a photoelectrochemical sensing technique that uses light to activate an electrochemical reaction at the surface of a semiconducting photoelectrode.^1,2^ Using LAES, it is possible to confine an electrochemical reaction to a microscopic portion of a macroelectrode using focused illumination,^3−7^ enabling high-density, individually addressed electrochemical measurements on a macroscopic substrate with a single electrode connection. High-density sensing strategies are advantageous for increasing the statistical power of measurements, performing imaging, and for performing trace analysis at sub-pM concentrations.^8^ Previously, high-density electrochemical measurements were only possible using micro- or nanoelectrode arrays fabricated with photolithography^8,9^ or using scanning electrochemical probe measurements (e.g., SECCM, SECM, SICM, etc.).^10−12^ Using light to perform these types of high-density measurements has several advantages over electrode arrays because pre-patterned electrode locations are not required and virtual electrode arrays with many elements can be created with only a single electrical connection, although each element in the array must be probed in series.^13^ These are also advantages for scanning probe measurements, but “switching” times between locations are often faster using light compared to scanning probes. A number of applications for LAES have been demonstrated including the development of (bio)sensors,^2^ imaging,^5,14^ surface patterning,^15−17^ and single-cell analysis.^14^ There are a number of LAES modes including those based on the electrochemical techniques of potentiometry, alternating current impedance, and direct current amperometry/voltammetry. Each mode has different requirements of the semiconductor/solution interface, as discussed in the recent review by Meng et al.^18^ In this article, we focus on LAES that involves Faradaic electron transfer between a semiconductor and a freely diffusing redox species in solution.

While other methods exist (reviewed in ref (13)), an appealing sensor configuration for LAES is to use a semiconductor/metal (SM) or semiconductor/insulator/metal (MIS) junction. In an idealized SM junction LAES, the semiconductor serves as the light absorber while the metal layer serves as the interface for electron transfer with the solution (i.e., the sensing layer).^19^ The difference in the work functions between the metal and the semiconductor induces favorable band bending in the semiconductor. Favorable band bending makes the sensor photoactive and establishes the photovoltage, which is the anodic or cathodic shift in effective redox potential observed in LAES voltammograms.^20^ Performing the electrochemical reaction on the metal surface often sidesteps some of the complications associated with semiconductor photoelectrodes (e.g., changing kinetics with differing redox potentials^21^). The metal layer also has the advantage of protecting the semiconductor from corrosion in aqueous electrolytes,^22^ even when the semiconductor is only partially covered with metal nanoparticles.^23^ Recently, our group demonstrated that n-type Si (n-Si) coated with electrodeposited Au nanoparticles (NPs) showed excellent electrochemical behavior that was stable for at least 1000 cycles.^24^ We used these sensors for measuring dopamine in buffer at sub-μM concentrations and created virtual arrays to get around issues of electrode fouling by catecholamine oxidation products. Gooding and co-workers showed that Au NPs attached to n-Si using a self-assembled monolayer were able to measure redox species over a fairly broad potential range (−0.25 to 0.25 V).^25^ Sojic, Loget, and co-workers employed MIS junctions to perform photoinduced electrogenerated chemiluminescence (ECL), which enables ECL at very low applied potentials and may lead to exciting new imaging strategies for biological samples.^26−31^

One major challenge for LAES using semiconductor/metal (as well as semiconductor/liquid) junctions is that only one electrochemical reaction (either oxidation or reduction) will be activated by light. When the electrochemical reaction of interest is an oxidation, n-type semiconductors are required, while p-type semiconductors are required for reductions. In order to be light addressable, the semiconductor must be in depletion, meaning that the concentration of minority carriers (i.e., holes for n-type or electrons for p-type semiconductors) is sufficiently low near the surface in the dark to kinetically inhibit charge transfer in one direction at the interface.^32^ In general, n-Si will be in depletion when paired with high work function metals (e.g.*,* Au and Pt), while p-Si will be in depletion when paired with low work function metals (e.g., Ti and W).^32^ Unfortunately, most of the most useful metals for electrochemical sensors (like Au and Pt) cannot be paired with p-type semiconductors to make LAES because their work functions are too high. It is obviously desirable to find a methodology that enables metals to be used for reductions on light-addressable electrochemical (LAE) sensors.

Square wave-voltammetry (SWV) is a differential pulse voltammetric technique that is widely used for electroanalysis, mechanistic studies, and measurements of heterogeneous electron transfer kinetics.^33^ In SWV, a square-wave potential pulse is applied over a step potential, and the current is sampled at the end of each pulse. By sampling the data at the end of the pulse, background charging currents are largely eliminated from the signal.^34^ The data are most often presented as a difference current (*i*_difference_ = *i*_forward_ – *i*_reverse_) versus the step potential. The difference current is amplified compared with currents measured using amperometry or cyclic voltammetry (CV) for reversible and quasi-reversible redox reactions.^35^ For irreversible reactions, the current is attenuated compared with CV.^36,37^ Surprisingly, there are no examples of SWV being used in combination with Faradaic LAE sensors. However, Zhang and co-workers recently combined SWV with LAE using field-effect devices constructed from electrolyte/insulator/semiconductor junctions.^38^ The sensors were used to measure pH and urea by capacitive currents induced by drift and diffusion of carriers within the semiconductor and subsequently to image patterned surfaces with micron-scale resolution.

Here, we show that by probing n-Si/Au LAE sensors using SWV, it is possible to achieve a light-activated response for both oxidations and reductions on a single LAE sensor. We tested the approach using the oxidation of ferrocene methanol (FcMeOH) and reduction of Ru(NH_3_)_6_^3+^ as model redox species. For Ru(NH_3_)_6_^3+^, we observed peaks consistent with an irreversible reduction in the dark. Upon illumination, the reaction becomes quasi-reversible (as confirmed by CV), and the SWV signal is drastically improved due to the amplification imparted by the change in reversibility. After studying the effects of the square-wave pulse and frequency, we observed a ∼41× increase in current under illumination compared with dark currents. In contrast, CV showed equal-magnitude dark and light currents under the same conditions. Using partially illuminated samples, we observed that the peak current increases with increasing illumination intensity, which we attributed to changes in carrier generation and minority carrier concentration. These results open up the possibility of two new avenues for LAES research. First, it enables a photoactive response for reductions on n-type semiconductors due to the pulsed nature of the waveform and the “switching” of the redox reaction from irreversible in the dark to reversible under illumination. Second, it should enable lower detection limits to be realized for LAES due to the suppression of background currents and open up new opportunities for LAES in trace analysis.

## Experimental Section

### Materials and Solutions

A detailed description of all materials and solutions is provided in Section S1 in the Supporting Information.

### LAES Sample Preparation

Si wafers were cleaned using isopropanol, ethanol, and water by sonication for 10 min each, with copious rinsing in between each solvent. The cleaned wafers were diced into ∼1.5 cm^2^ samples by scoring the unpolished side with a diamond tipped pen and breaking the wafer along the scratch line. Each sample was rinsed with water and dried using compressed air to remove the dust generated during the cleaving. Next, each sample was cleaned with Piranha solution (a 3:1 mixture of concentrated H_2_SO_4_ to 30% H_2_O_2_) for 30 min at 105 °C. *Caution: Piranha solution reacts violently with organic materials.* The samples were thoroughly rinsed with 18.2 MΩ•cm DI water before being submerged in a 40% NH_4_F solution (previously de-oxygenated with Ar for >30 min) for 10 min to remove the oxide on both sides of the sample. H-termination was confirmed by placing a small droplet of water on the surface to ensure that the sample was hydrophobic. Ohmic back contacts were prepared by contacting a copper wire to the unpolished side of the wafer with indium solder. The sensors were insulated by sealing the entire assembly in 3M electroplater’s tape, which included a 2 or 3 mm opening that allowed exposure of the polished front Si surface to the electrolyte. The opening in the electroplater’s tape was cut using a Glowforge laser cutter. This stage of sample preparation was done in batches of 10–20 electrodes. The samples were stored in the dark until further use.

Electrodeposition of Au NPs on the surfaces of Si was performed using a modified protocol from Allongue et al.^39^ and shown schematically in Figure S1. The exposed Si surface was etched in de-oxygenated 40% NH_4_F for 10 min to remove oxide formed during storage. The electrode was rinsed with copious amounts of DI water and immersed in an electrodeposition solution consisting of 0.5 mM HAuCl_4_, 1 mM KCl, 0.1 M K_2_SO_4_, and 1 mM H_2_SO_4_ for 5 min. In order to prevent oxidation, each electrode was biased at −1.9 V vs Ag/AgCl before being dipped in the deposition solution. Note that for the electrodeposition experiments, a graphitic carbon rod was used as the counter to prevent Pt contamination of the LAES. The deposition was carried out with room lights on but without direct illumination of the semiconductor surface.

### (Photo)electrochemical Measurements

(Photo)electrochemical experiments were performed using either a CH Instruments 760E or a HEKA ELP-1 bipotentiostat. All measurements were performed in a 30 mL electrochemical cell with a borosilicate glass window in a three-electrode configuration. A saturated calomel electrode (SCE) and a Pt wire were used as the reference and counter electrodes, respectively. Illumination of the semiconductor was performed using a white light LED from AM Scope with a calibrated intensity of 85 mW cm^–2^. For varied power experiments, the semiconductor was illuminated with a 530 nm (2.3 eV) fiber-coupled LED (M530F2) coupled to a 550 μm diameter fiber optic cable (MHP550L02, Thorlabs; 0.22 NA), a F240SMA-532 collimator, and a 20× LWD objective (Mitutoyo M Plan Apo; 0.42 NA). The LED intensity was controlled using a constant-current LED driver from Thorlabs (UPLED) controlled using upSERIES software. A calibration curve relating the LED driver current and LED intensity was created by measuring the intensity using a USB power meter from Thorlabs (PM16-122) and is shown in Figure S6 in the Supporting Information. Prior to each set of experiments, a one-point power calibration was performed. All optical components were purchased from Thorlabs and housed inside a custom-built dark box to eliminate ambient light.

### Energy-Dispersive X-ray Spectroscopy

Energy-dispersive X-ray spectroscopy (EDX) was performed using a Hitachi S-3400N SEM in secondary electron mode using a 30 kV accelerator voltage.

## Results and Discussion

### Characterization of n-Si/Au NP LAES

We performed detailed characterization of the LAES samples using SEM/EDX and electrochemical impedance spectroscopy, as described in the Supporting Information, Section S2. In summary, the sensors were partially coated with gold and had a flat band potential (*E*_fb_) around −0.8 V vs SCE—slightly cathodic compared with our previous study, which used 0.1 mM HAuCl_4_ for electrodeposition.^24^ A schematic band diagram is also presented in the Supporting Information, Section S2.

### CV of FcMeOH and Ru(NH_3_)_6_^3+^ with n-Si/Au NP LAE Sensors

**Figure 1:**
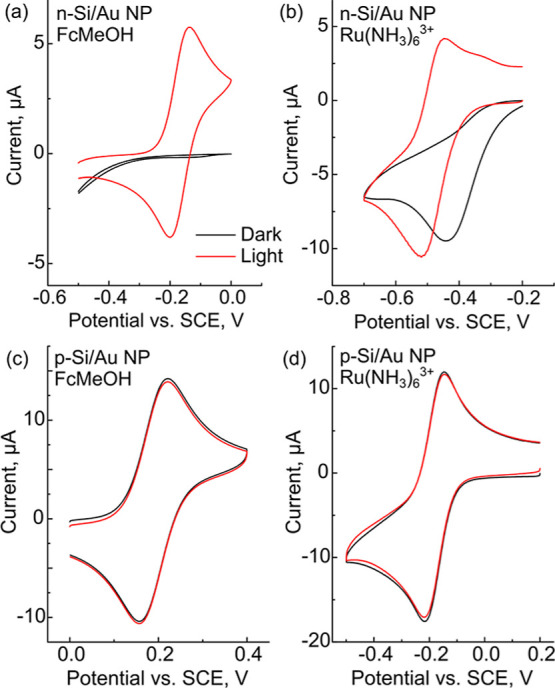
Cyclic voltammograms of 1 mM (a) FcMeOH and (b) Ru(NH_3_)_6_^3+^ in 0.1 M KNO_3_ with n-Si/Au NP LAES. Cyclic voltammograms of 1 mM (c) FcMeOH and (d) Ru(NH_3_)_6_^3+^ in 0.1 M KNO_3_ with p-Si/Au NP LAES. In all plots, the black traces were collected in the dark, and the red traces were illuminated with 85 mW cm^–2^ white light. Experimental conditions: *v* = 0.1 V s^–1^; reference = SCE; counter = Pt wire.

We performed CV in order to characterize n-Si/Au NP LAES using both oxidation and reduction reactions. We chose the oxidation of FcMeOH and the reduction of Ru(NH_3_)_6_^3+^ as model redox reactions because they have fast heterogeneous electron transfer (HET) kinetics, have redox potentials more positive than *E*_fb_, and demonstrate how the initial redox reaction (oxidation vs reduction) impacts the observed electrochemistry. Figure 1a shows that in the dark (black trace), FcMeOH displays very little electrochemical activity, with relatively small cathodic currents flowing at potentials less than −0.3 V. We attribute these small cathodic currents to oxygen reduction, as the solutions were not de-aerated prior to the experiments. The CVs of illuminated FcMeOH (red trace) show a well-defined diffusional response with Δ*E*p ≈ 63 mV and a peak current ratio (*i*_p,a_/*i*_p,c_) close to one. Figure 1b shows that for Ru(NH_3_)_6_^3+^ in the dark (black trace), the cathodic scan shows a large irreversible peak. Upon illumination (red trace), the cathodic peak shifts approximately −78 mV and an oxidation wave emerges. The Δ*E*p for Ru(NH_3_)_6_^3+^ under illumination was ≈72 mV, and the peak current ratio was also close to 1. These results show that although n-Si/Au NP LAES show fast HET for both FcMeOH and Ru(NH_3_)_6_^3+^ under illumination, the Ru(NH_3_)_6_^3+^ has significant cathodic dark currents.

The differences in the electrochemical behavior of the two redox molecules on n-Si/Au NP LAES are a direct result of the semiconductor being in depletion—meaning there are insufficient concentrations of minority carriers (holes for n-type semiconductors) but an excess of majority carriers (electrons for n-type semiconductors).^40^ FcMeOH is unable to be oxidized in the dark on the first sweep of the CV because the oxidation requires holes. On the subsequent cathodic sweep, there is no reduction wave observed because FcMeOH^+^ is not generated on the positive scan. Once illuminated with photons having energy greater than the band gap, electron/hole pairs are formed, which increases the minority carrier concentration and enables FcMeOH oxidation to occur on the anodic scan. On the illuminated cathodic sweep, there are sufficient majority carriers in n-Si (electrons) to drive the reduction. For the Ru(NH_3_)_6_^3+^ case, the molecule is initially present in its oxidized form, so on the initial cathodic sweep in the dark, Ru(NH_3_)_6_^3+^ can be reduced to Ru(NH_3_)_6_^2+^ because there are a sufficient number of electrons available for reduction. However, on the anodic scan in the dark, no current flows because of the low minority carrier concentration. Once illuminated, the cathodic peak potential shifts, and the oxidation of Ru(NH_3_)_6_^2+^ is enabled on the anodic scan. The origin of the photovoltage shift in both FcMeOH and Ru(NH_3_)_6_^3+^ is likely caused by the semiconductor/metal junction.^19^ These results are consistent with our previous study^24^ and results from Gooding and co-workers where Au NPs were attached to Si surfaces through monolayer chemistry.^25^

The particular reactivity of depleted semiconductor electrodes has important consequences for LAES. The most obvious is that n-type semiconductors cannot be used to study reductions without a high background dark current using CV because the background current will be of similar magnitude to the signal current. In order to study reductions with LAES, p-type semiconductors are used.^2^ Unfortunately, many of the most electrochemically useful metals (e.g., Au, Pt, etc.) will not deplete p-type semiconductors because the metal’s Fermi level is too close in energy to the Fermi level of the semiconductor.^19^ To validate this with our experimental configuration, we prepared LAES samples using lowly doped p-Si and Au NPs using a similar procedure to the n-Si samples. The only difference is that p-Si was illuminated during electrodeposition because the minority carriers in p-Si electrons are not present in sufficient concentrations to drive the electrodeposition of Au. Figure 1c shows CVs of FcMeOH using p-Si/Au NP LAES, and clear quasi-reversible redox peaks were observed under dark and illuminated conditions. Figure 1d shows CVs of Ru(NH_3_)_6_^3+^ using p-Si/Au NP LAES, showing similar behavior to the FcMeOH data. These data experimentally demonstrate one of the major limitations of using SM junctions for LAES: some of the most useful metals for electrochemical sensing (e.g., Au) cannot be used to create LAES for reductions when paired with p-Si. Therefore, alternative strategies must be employed to study reductions with SM junctions.

### SWV with LAES under Full Illumination

**Figure 2:**
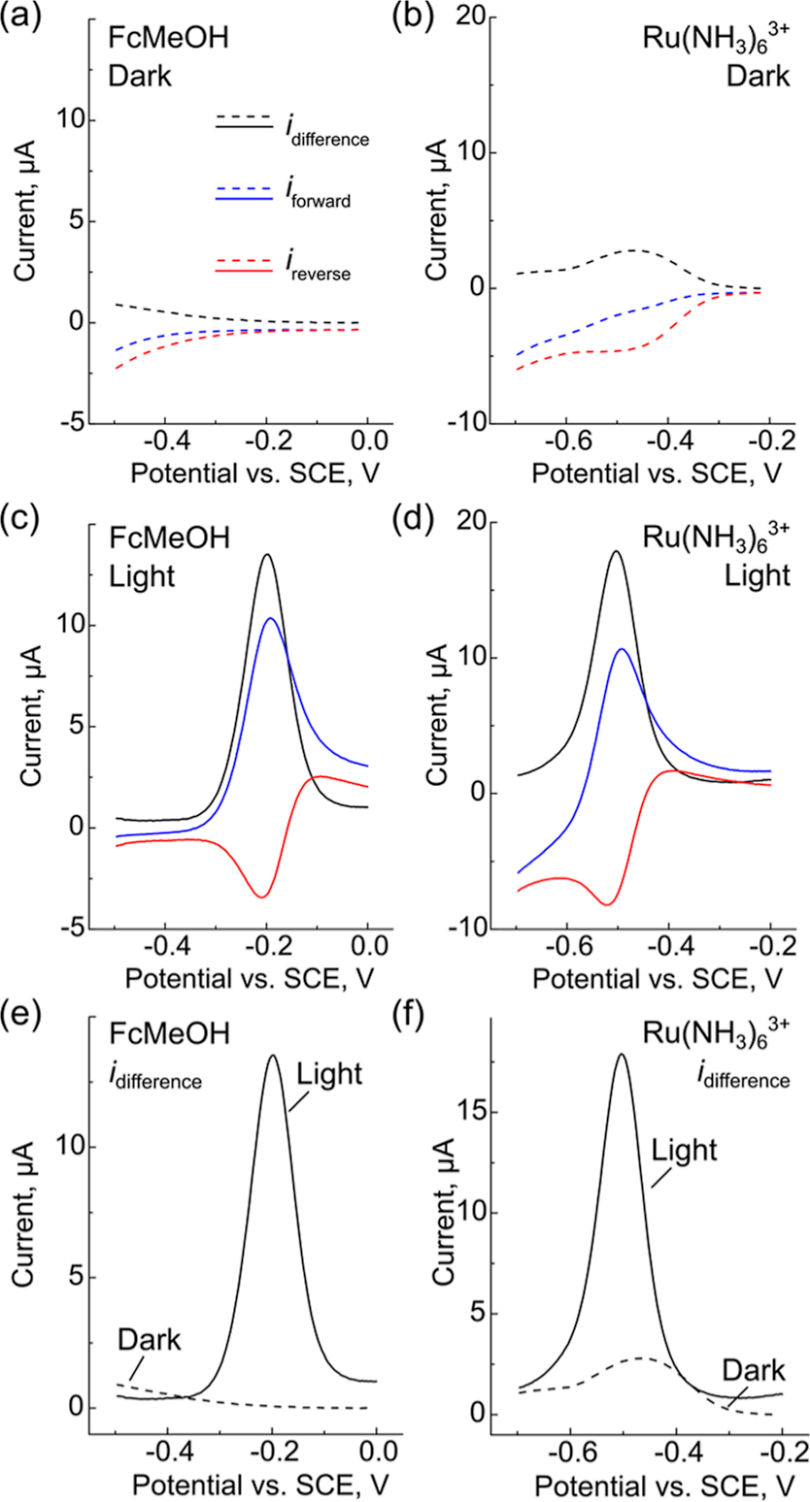
SWVs showing the forward (blue), reverse (red), and difference (black) currents for (a,c,e) FcMeOH and (b,d,f) Ru(NH_3_)_6_^3+^ in the dark (a,b), light (c,d), and comparison of the light/dark difference currents (e,f). SW voltammograms were collected with a 25 mV amplitude and a 15 Hz frequency.

We hypothesized that interrogating LAES with SWV might enable measurement of reductions with n-Si/Au LAE sensors because the CVs of Ru(NH_3_)_6_^3+^ in the dark were irreversible but near-reversible under illumination. We hypothesized that this condition would lead to the current being amplified under illumination but attenuated in the dark because the diffusion layer is not refreshed between pulses.^35,36,41^ For a brief review of SWV and the potential waveform, see Section S4 in the Supporting Information. We initially investigated the forward (blue traces), reverse (red traces), and difference currents (black traces) for FcMeOH oxidation and Ru(NH_3_)_6_^3+^ reduction in the dark (dotted lines). For FcMeOH oxidation, SWV was carried out in “normal mode”, where an anodic scan was used to probe an oxidation reaction. For Ru(NH_3_)_6_^3+^ reduction, SWV was carried out in “reverse mode” where an anodic scan was used to probe a reduction.^37,42,43^Figure 2a shows the dark traces for FcMeOH oxidation. The forward and reverse currents are cathodic and small at potentials less than −0.3 V vs SCE, likely due to oxygen reduction. Because these traces are comparable in magnitude, the overall difference current is near-zero. Similar to the CVs in Figure 1a, FcMeOH is not oxidized in the dark due to the lack of minority charge carriers, and the reverse reaction is impossible without the oxidized product. As a result, very small currents are observed in the forward, reverse, and difference traces. For Ru(NH_3_)_6_^3+^ in the dark (Figure 2b), the forward currents (blue dash) show a sloping background current, which flattens out ca. −0.34 V vs SCE. In contrast to the FcMeOH results, the reverse currents (red dash) show a cathodic peak centered at −0.47 V vs SCE resulting from the reduction of Ru(NH_3_)_6_^3+^ during the reverse pulses. The difference current (black dash) shows a positive peak resulting from the subtraction of the forward and reverse peaks. This attenuated peak will likely exist for all reduction reactions studied on n-type semiconductors. Figure 2c,d shows that once illuminated, the FcMeOH and Ru(NH_3_)_6_^3+^ forward, reverse, and difference currents are all consistent with diffusion-limited responses on metallic electrodes. The changes in the SW voltammograms originate from the charge carriers generated by illumination with light with energy greater than the band gap energy. Figures 2e,f compares the difference currents for the dark and light traces. In both cases, they show dramatic enhancement of the signal for both redox species upon illumination. For FcMeOH, there is a true “on/off” response for the dark and light traces. For Ru(NH_3_)_6_^3+^, the light current is ∼8.6× larger than the dark current. Overall, the dark current behavior is qualitatively similar to reverse scan SWV with an irreversible redox species, while the illuminated traces are similar to a reversible redox species.

In order to see if these results were limited to simple redox reactions, we performed similar measurements with methylene blue (MB). MB is a widely used electrochemical label, often used for aptamer-based sensors as well as other applications.^44,45^ MB has a redox potential in the range of −0.29 V vs SCE^46^ and should display light addressable behavior based on *E*_fb_. Figure S5 displays dark and light CVs for 10 μM MB in 1× PBS on n-Si/Au NP LAES and shows a behavior very similar to Ru(NH_3_)_6_^3+^. In the dark, a single reduction wave is present at −0.35 V vs Ag/AgCl. Once illuminated, the reduction peak shifts 150 mV negative, and an oxidation peak appears. The peak separation is sub-Nernstian and around 25 mV, which suggests some adsorption of the molecule to the sensor surface. Figure S5b shows light/dark SW voltammograms of the same solution collected with the same sensor as Figure S5a. A large Gaussian wave is observed that shows a significant increase in peak current compared to the dark current sample, demonstrating that SWV can be used when studying more complex reductions.

There are several interesting implications from these results. First, they demonstrate that Faradaic LAES are compatible with SWV. The direct consequence of this is that analytical applications of LAES should benefit from the improvement in sensitivity and detection limits afforded by SWV. Second, the data show that it is possible to achieve a light-activated response for both oxidations and reductions using an n-type semiconductor. This is enabled by the potential pulse sequence, differential current measurement, and the rectifying properties of the SM junction, which make an electrochemically reversible reaction behave irreversibly under dark conditions. This observation is significant because MS junction LAE sensors require a favorable energy difference between the semiconductor and metal to place the semiconductor into depletion and generate a photovoltage. For p-type Si, which is the semiconductor of choice for reductions, only metals with relatively low work functions (e.g., W) are able to place the semiconductor into depletion.^47^ As shown in Figure 1c,d, when Au was paired with p-Si, no photoactivity was observed. Given that Au and other high work-function metals are some of the most widely used materials in electroanalysis, it is desirable to integrate these materials into LAES for reduction reactions.

### Effect of Step Amplitude and Frequency

**Figure 3:**
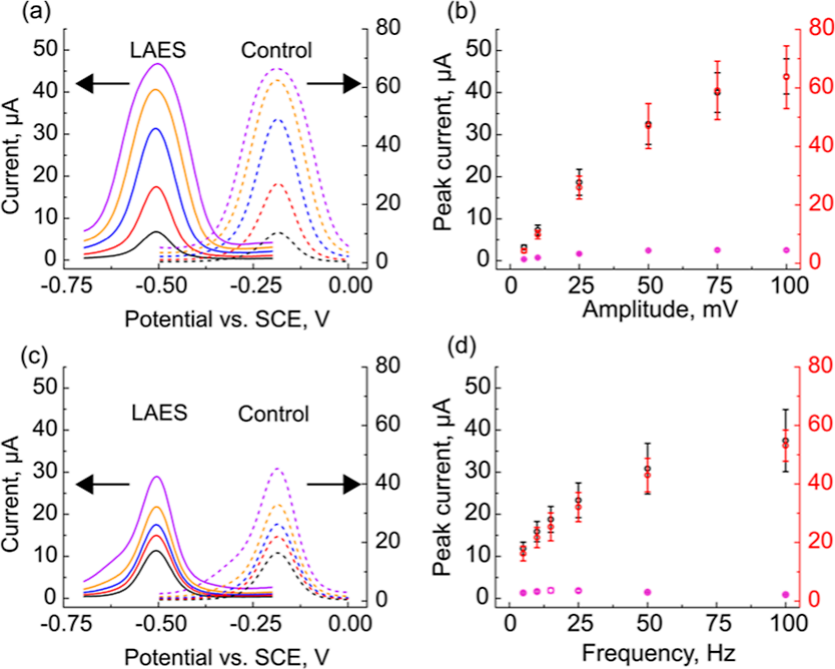
(a) Representative SWVs of 1 mM Ru(NH_3_)_6_^3+^ with illuminated nSi/Au NP LAES (solid lines) and p^+^Si/Au NP control samples (dashed lines) collected with *f* = 15 Hz and amplitude 5–100 mV; (b) plot of *i*_p_ versus amplitude; (c) representative SWVs of 1 mM Ru(NH_3_)_6_^3+^ with illuminated nSi/Au NP LAES (solid lines) and p^+^Si/Au NP control samples (dashed lines) collected with amplitude = 25 mV and *f* from 5 to 100 Hz; (f) plot of *i*_p_ versus *f*. For parts (b,c,e,f) the violet circles are nSi/Au NP LAES in the dark, black circles are the illuminated LAES, and the red circles are p^+^Si/Au NP controls. Error bars represent one standard deviation of three independently prepared samples.

We next investigated how the half-peak width, *w*_1/2_, and peak height, *i*_p_, would change in response to variations in the SW amplitude and frequency while holding the step size constant (Figure 3). Specifically, we were interested to see if we could further attenuate the dark current signal for Ru(NH_3_)_6_^3+^ reduction by simply changing the square-wave parameters.

Figure 3a,c shows representative SW voltammograms for Ru(NH_3_)_6_^3+^ on illuminated nSi/Au NP LAE sensors (solid lines) and non-photoactive p^+^-Si/Au NP controls (dashed lines). The SWVs in Figure 3a were recorded at varying amplitudes (range: 5–100 mV) with the frequency held constant at 15 Hz, while the SWVs in Figure 3c were recorded with varying frequencies (range: 5–100 Hz) with a constant amplitude of 25 mV. Representative dark current traces are shown in Figure S8 in the Supporting Information. Qualitatively, the data in Figure 3a,c show that the peaks broaden and the peak current increases as the SW amplitude and frequency increase, similar to metallic electrodes.^48^ The shape and behavior of the LAES and control samples are qualitatively similar. Note that these experiments were performed using electrodes with different diameters (2 vs 3 mm diameter disks for the LAES and control samples, respectively), so the currents are different. The major difference between the LAES and controls is the peak potential, *E*p, which is ca. −0.55 V for the LAES and −0.22 V for the control samples. The metallic control samples are centered near the formal potential of the redox couple (*E*° ≈ −0.22 V vs SCE). The cathodic shift is caused by the photovoltage generated by the SM junction. The similarity of these data suggests that carrier generation and transport in the SC do not play a significant role in the observed SWVs under the studied illumination conditions and that the observed current response is most likely controlled by the metal NP/solution interface rather than the semiconductor/solution interface.

Figure 3b,d shows how *i*_p_ changes with varying SW amplitude and frequency, respectively. For n-Si/Au NP samples in the dark, the peak height was virtually constant and very low over the entire range for both amplitude and frequency (violet circles). For the illuminated LAES (black circles) and metallic control samples (red circles), the peak height increased over the amplitude range from 5 to 50 mV before leveling off around 40 and 60 μA, respectively (Figure 3b). The illuminated (black circles) and control samples (red circles) showed an increasing peak current that was linearly dependent on *f*^1/2^ (*R*^2^ > 0.98 for both samples; Figure S9), suggesting that the system is diffusion controlled (Figure 3d). The impact of SW amplitude and frequency on *w*_1/2_ is less dramatic than peak current (Figure S10). In the dark, *w*_1/2_ varies between 150 and 200 mV for all measured amplitudes and frequencies. Under illumination, the sensors are closer to reversible at low amplitude (<25 mV) and low frequency (<25 Hz). A full discussion of these data is presented in the Supporting Information, Section S5.

We performed similar experiments using FcMeOH and found results that are consistent with Ru(NH_3_)_6_^3+^. The notable difference is that the FcMeOH dark traces are featureless because the forward reaction is blocked in the dark, as shown in Figure 2a. These data are presented in the Supporting Information, Section S6.

**Figure 4:**
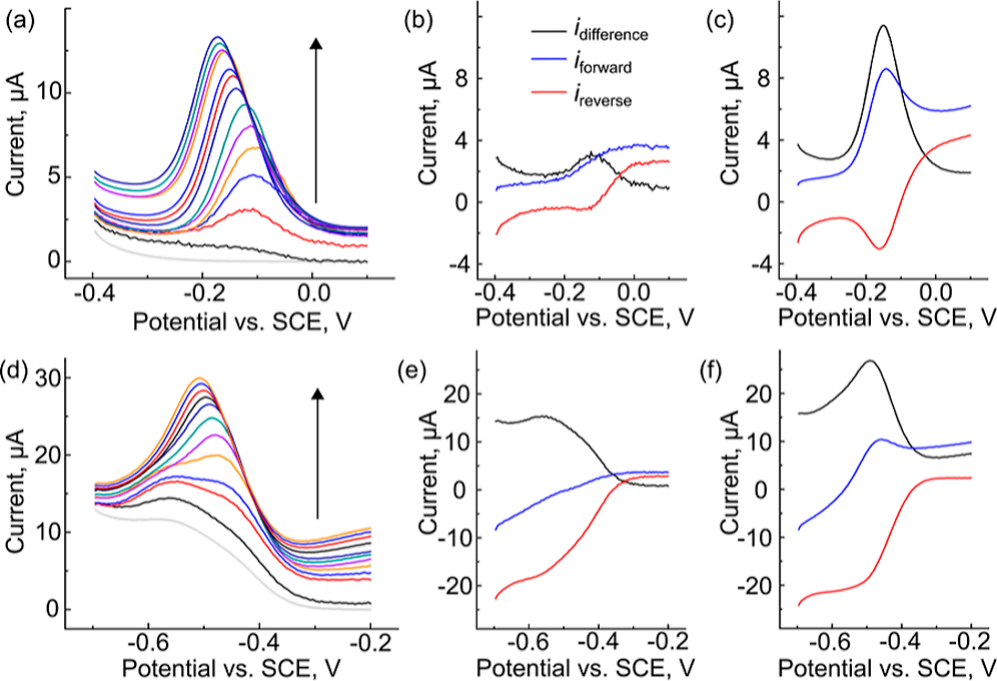
Effect of light intensity on illuminated LAES probed with SWV. (a) Representative SW voltammograms for the oxidation of FcMeOH in 0.1 M KNO_3_. (b,c) Forward, reverse, and difference current SW voltammograms collected high and low in illumination intensity, respectively. (d) Representative SW voltammograms for the oxidation of Ru(NH_3_)_6_^3+^ in 0.1 M KNO_3_. (e,f) Forward, reverse, and difference current SW voltammograms collected at high and low in illumination intensity, respectively. Frequency = 100 Hz, amplitude = 25 mV.

The important takeaway from the data in Figure 4 is that it is possible to improve the signal-to-background of these sensors by changing the square-wave parameters. For instance, at 10 Hz (amplitude = 25 mV), the illuminated signal is ∼9.6× larger, while at 100 Hz, the signal is ∼41× larger. Similar trends are observed with changing the amplitude, although the effect is less dramatic. These results are important for reductions studied with n-Si/Au NP LAES because there will always be a significant dark current background originating from the high electron concentration in the n-Si. Additionally, these results demonstrate that it may be possible to probe the kinetics of charge transfer at both illuminated and dark LAES using SWV due to the changes in *w*_1/2_.^48^

### Effect of Illumination Intensity on Partially Illuminated LAE Sensors

We next sought to understand how SWV would impact LAE sensors when they are partially illuminated with varying amounts of power. This type of experimental condition is used when imaging^13,18,49^ and performing array-type measurements^2,4^ with LAE sensors. Under these conditions, the dark currents are proportional to the electrolyte contact area, while the illuminated currents will be proportional to the illuminated area. In these experiments, we used a fiber-coupled 530 nm green LED that was collimated and focused through a lens to illuminate the surface of the semiconductor. The beam size was ∼1 mm in diameter, and the area of the electrode exposed to solution was 6 mm in diameter. Note that the minority carrier diffusion length is long for the high-quality crystalline Si used here, so the “effective” illuminated area is larger than ∼1 mm because the carriers can diffuse from the illumination area outward toward the solution and react with the redox species, as discussed below.

Figure 4a,d shows SW difference voltammograms for the oxidation of FcMeOH and Ru(NH_3_)_6_^3+^, respectively, on nSi/Au NP LAES illuminated with a 530 nm LED over the power range from ∼0 to 1 mW. A dark current background-subtracted data set for Ru(NH_3_)_6_^3+^ is included in Figure S14 in the Supporting Information. For FcMeOH (Figure 4a), the initial dark scan (gray trace) shows very little anodic current at potentials more positive that −0.2 V vs SCE. However, for Ru(NH_3_)_6_^3+^, the dark trace shows a broad wave spanning the potential range from approximately −0.6 to −0.35 V vs SCE. This wave corresponds to the irreversible dark current reduction of Ru(NH_3_)_6_^3+^. We note that dissolved oxygen may also contribute to the dark current signal, as the solutions were not de-aerated prior to the experiments. For both redox species, the peak current increases with increasing power before leveling off at a stable value, as shown in Figure S15a,c for FcMeOH and Ru(NH_3_)_6_^3+^, respectively. We also observed changes in the *w*_1/2_ and *E*p with increasing illumination intensity (Figure S15b,d). For FcMeOH, *w*_1/2_ decreases initially, reaching a minimum value of ∼100 mV at 0.172 mW before steadily increasing to ∼120 mV at 0.85 mW (Figure S15b). For Ru(NH_3_)_6_^3+^, the *w*_1/2_ at low power is predominantly influenced by the irreversible wave and is thus very broad, reaching a maximum value of ∼205 mV at 0.065 mW before decreasing to ∼130 mV at *P* > 0.5 mW (Figure S15d). The *E*p has a cathodic shift of −180 mV for FcMeOH and −50 mV for Ru(NH_3_)_6_^3+^ (Figures S15b,d).

To understand these trends more deeply, we investigated the forward (blue traces), reverse (red traces), and difference currents (black traces) for FcMeOH and Ru(NH_3_)_6_^3+^. At low intensity (0.038 mW; Figure 4b), the forward current is sigmoidal in FcMeOH, while the reverse current displays a small cathodic peak, suggesting that there is a change in the “effective” area of the electrode for the forward (oxidation) and reverse (reduction) scans. The sigmoidal current is likely limited by carrier generation and collection rather than mass transfer, given the relatively large illumination area. At higher intensities (0.308 mW; Figure 4c), the forward and reverse traces both show evidence of diffusion-limited voltammetry, similar to the data in Figure 2c collected with 85 mW cm^–2^ white light that illuminated the entire electrode surface. For Ru(NH_3_)_6_^3+^, the potential at the start of the scan is sufficient to reduce the Ru(NH_3_)_6_^3+^ initially present in solution, leading to cathodic currents flowing for both the forward and reverse pulses. At low intensity (0.038 mW, Figure 4e), the forward currents transition from cathodic to anodic at −0.5 V vs SCE and reach a steady-state current of ∼3 μA at potentials more positive than −0.3 V vs SCE, which is a similar value to FcMeOH, supporting that the oxidation currents are limited by light at low intensities. The reverse and difference currents show a peak. At higher intensities (Figure 4f), forward, reverse, and difference currents show diffusion-limited behavior.

The data in Figure 4 suggest there are two factors that lead to increases in current with increasing power. The first is that at low power, the forward current response is limited by carrier generation and collection rather than the mass transport of reactants and products. Several studies have recently shown there is a “transition zone” where enough carriers are generated in order to support the number of molecules generated.^4,50^ Once above this intensity, the forward current is limited by mass transfer rather than illumination intensity. In these measurements, we estimate the cutoff power to be ∼51.5 μW, which is the first power to show diffusion behavior on the forward current SW voltammograms in Figure S16. The second factor is that the effective area of the electrode increases with increasing power because more minority carriers are generated at higher intensities.^32^ When carriers are generated locally, they are able to diffuse away from the illumination area, as recently imaged using carrier generation-tip collection SECCM by Hill and Hill.^51^ For high-quality crystalline Si, the minority carrier diffusion length is significant and depends on the Si doping level and crystal quality (reasonably estimated to be ∼100 μm; ref (52)). At lower intensity, fewer holes are generated, and they are rapidly transported to the interface to react with the FcMeOH or Ru(NH_3_)_6_^2+^ (or will recombine in the semiconductor). At higher intensities, more holes are generated and can diffuse further from the illumination spot, ultimately reacting at the interface (or recombining within the SC bulk) and leading to an increase in the effective electrode area. At low intensity, the reverse currents show peaks, supporting our hypothesis that the area available for reductions is larger and not dependent on illumination. Interestingly, although the entire electrode surface is available for reductions, we still observe well-defined SW voltammograms for partially illuminated samples in Ru(NH_3_)_6_^3+^. This is because the oxidation is confined to the illumination spot (plus minority carrier diffusion length), and so the amplification of the SWV signal is confined to this location.

## Conclusions

While LAES has tremendous potential for creating high-density electrochemical measurements, forming virtual arrays, and imaging biochemical processes in vitro, one drawback is that LAE sensors are only photoactive for one electrochemical reaction depending on the type of semiconductor used for light absorption: oxidations on n-type materials and reductions on p-type materials. In this contribution, we showed that using SWV with LAE sensors enables both oxidations and reductions to be studied with a single sensor. We studied the oxidation of FcMeOH and the reductions of Ru(NH_3_)_6_^3+^ and MB on n-Si/Au NP LAE sensors. For oxidations on an n-type LAE sensor, SWVs had the expected on and off responses under illumination and in the dark, respectively. For reductions on an n-type LAE sensor, dark SWVs showed a peak in the dark current scan, attributed to the reduction on the reverse pulse. While this signal cannot be completely eliminated, we observed a 41× increase in signal after illumination at high SW frequency. This technique is also compatible with partially illuminated LAE sensors.

We expect the results here to have an impact in the following ways. First, SWV enables trace measurements by decreasing the background current associated with the charging of the double layer. As a result, we expect direct improvements in detection limits for voltammetric LAE sensors. Second, these results are especially useful for semiconductor/metal LAE sensors because many metals useful for electroanalysis are incompatible with p-type Si and cannot be used for photoactive reductions. Third, recent results have shown that SWV is especially powerful when the entire current versus time (*i*–*t*) profile is recorded.^34,53^ Cobb and Macpherson showed that the current decay in the initial portion of the pulse can be used to measure solution resistance and conductivity, which can be used to determine the solution conductivity.^34^ Abeykoon and White showed that by recording the continuous *i*–*t* curve, multiple-frequency SW voltammograms can be extracted by sampling the current at different time points after the potential pulse. These multiple-frequency experiments can be used to measure the electron transfer kinetics of freely diffusing and surface-bound redox species. We expect that probing semiconductor photoelectrodes with continuous SWV may enable simultaneous measurement of both capacitive (i.e., flat band potentials) and Faradaic effects (i.e., charge transfer). Finally, we would like to acknowledge the limitations of the methodology presented here. In order to observe “light-on” responses for reductions on n-type semiconductors, the redox reaction must be quasi-reversible. In cases where the reduction is irreversible, we do not expect to observe a significant change between the dark and light traces.

## Supporting Information Available

Calibration of local LED power; additional characterization of n-Si/Au NP LAES; impact of SW amplitude and frequency on dark current for Ru(NH_3_)_6_^3+^; impact of SW amplitude and frequency on FcMeOH; and additional analysis of local illumination SWV measurements (PDF)
