# Supplementary material for: Square-Wave Voltammetry Enables Measurement of Light-Activated Oxidations and Reductions on n-Type Semiconductor/Metal Junction Light-Addressable Electrochemical Sensors
Source: Anal Chem. 2023 Jun 5;95(24):9219–26. doi: 10.1021/acs.analchem.3c00630 (PMC10285716; doi:10.1021/acs.analchem.3c00630)
Supplement: Supplementary file 1 — ac3c00630_si_001.pdf [file ac3c00630_si_001.pdf]

Supporting information

Square wave voltammetry enables measurement of light-activated oxidations and reductions on n-type semiconductor/metal junction light addressable electrochemical sensors

Enock G. Arthur[†], Hana Ali[†], Armeen Hussain[†,] and Glen D. O'Neil[†‡*]

[†]Department of Chemistry and Biochemistry, Montclair State University, Montclair, NJ 07043

[‡]Sokol Institute for Pharmaceutical Life Sciences, Montclair State University, Montclair, NJ 07043

*To whom correspondence should be addressed: oneilg@montclair.edu

---

Table of contents

Section	Page
S1. Materials and solutions	S2
S2. Fabrication and characterization of LAE sensors	S2
S3. Calibration of local LED power	S5
S4. Brief description of square wave voltammetry	S5
S5. Impact of SW amplitude and frequency on dark current for $Ru(NH_3)_6^{3+}$	S6
S6. Impact of SW amplitude and frequency on FcMeOH	S7
S7. Additional analysis of local illumination SWV measurements	S10
S8. References	S11

Section S1. Materials and solutions

Ferrocene methanol (FcMeOH; 97%) and hexaammineruthenium(III) chloride ($Ru(NH_3)_6^{3+}$; 98%) were from Acros Organics, hydrogen tetrachloroaurate(III) trihydrate ($HAuCl_4$•H_2O; 99.99%) was from Alfa Aesar, ammonium fluoride (40% m/m; semiconductor grade) was from Honeywell, and high purity argon was from AGL (Clifton, NJ). All other chemicals were from Fisher, of ACS reagent grade or better, and used without purification. Stock solutions of 2.0 mM FcMeOH were prepared in 0.1 M KNO_3 and sonicated for at least one hour to help dissolve the redox couple. Once dissolved, the stock solutions were filtered with a 0.2 µm polycarbonate filter and stored at 4 ºC. Sample solutions were prepared daily by diluting the stock solution with 0.1 M KNO_3. $Ru(NH_3)_6^{3+}$ solutions were prepared fresh daily using 0.1 M KNO_3 as the supporting electrolyte. All solutions were prepared using deionized water with resistivity of 18.2 MΩ·cm at 22±2 °C purified using a benchtop Millipore Simplicity system.

LAE sensors used in this study were prepared using lowly-doped n-type Si (100), p-type Si (100), and highly-doped (metallic) p^+-Si (100) from Pure Wafer (San Jose, CA). All wafer types were single-side polished and 500-550 µm thick. The n-type wafers were doped with phosphorous (resistivity 1-5 Ω•cm), the p-type wafers were doped with boron (resistivity 1-5 Ω•cm), and the p^+-type wafers were doped with boron (resistivity <0.005 Ω•cm). Semiconducting Si has a band gap $E_g \approx 1.1$ eV (≈1100 nm).

Section S2. Fabrication and characterization of LAE sensors

In our previous work, we demonstrated that n-type Si coated with electrodeposited Au NPs behave as LAES with nearly reversible electrochemistry towards a number of redox species and excellent stability over 1000 CV cycles.[1] Here, we use this platform to study the square wave voltammetry of two outer-sphere redox species: ferrocene methanol (FcMeOH) and ruthenium (III) hexamine ($Ru(NH_3)_6^{3+}$). We prepared the samples by electrodepositing Au on freshly-etched n-Si, following earlier work from Allongue et al. (Fig. S1).[2] In order to confirm the presence of Au on the n-Si, we performed EDX mapping (Fig. S2), which demonstrated successful Au deposition and partial coverage of the n-Si.

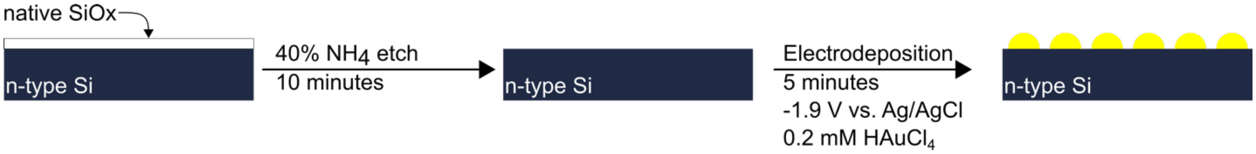

Figure S1: Fabrication of the nSi/Au NP LAES

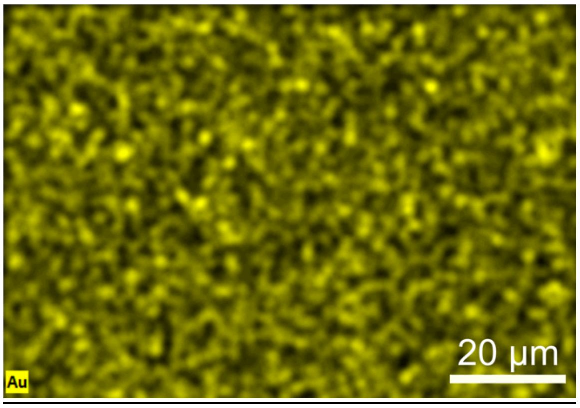

Figure S2: Energy-dispersive X-ray spectroscopy (EDX) map of n-Si/Au photoelectrode for Au. Images were obtained using a Hitachi S-3400N SEM in secondary electron mode using a 30 kV accelerator voltage.

We confirmed that the junctions were rectifying over the desired potential range by performing Mott-Schottky measurements (Fig. S3). We observed that the flat band potential, E_{fb}, was ca. –0.8 V vs. SCE, suggesting that the semiconductor would be in depletion (and hence photoactive) at potentials more positive than this value. We note that Mott-Schottky measurements on heterogeneous samples are difficult to interpret and can lead to significant uncertainties in the flat band potential.[3] However, the voltammetry data below confirm the semiconductor to be in depletion. In summary, physical and EIS characterization of these samples was consistent with the our previously published sensors.[1] However, the flat band potential of these sensors was ~200 mV more cathodic than our previous study, likely because of the increased concentration of $AuCl_4^-$ in the electrodeposition bath.

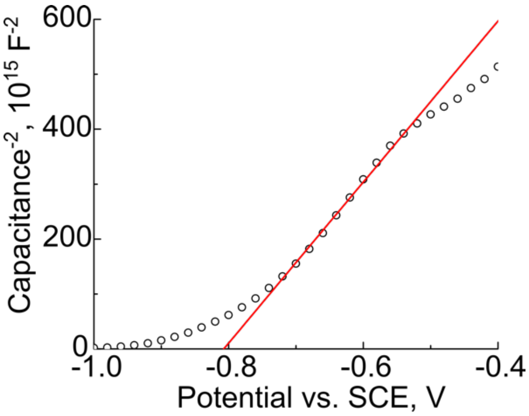

Figure S3: Mott-Schottky characterization of n-Si/Au NP LAES. EIS measurements were carried out with 50 kHz frequency over the range from -1 to 0 V vs. SCE. The electrolyte was 1 mM FcMeOH with 0.1 M KNO_3 supporting electrolyte. Reference: SCE; counter: Pt wire.

Based on the estimated flatband potential, we were able to draw a schematic band diagram for the metal/semiconductor junctions used herein (Fig. S4). For clarity, we have also included the approximate redox potentials of the three redox species used. As discussed above, E_{fb} is approximately –0.8 V vs. SCE. The conduction band edge (E_{cb}) is estimated to be –1.05 V vs. SCE using equation S1,

$$E_{cb} = E_{fb} + k_B T ln\left(\frac{N_d}{N_c}\right) \quad \text{(S1)}$$

where k_B is Boltzmann's constant (= 8.62•10^{-5} eV K^{-1}), T is the absolute temperature (= 293 K), N_d is the bulk dopant density (= 1.5•10^{15} cm^{-3}, estimated based on the manufacturer's quoted resistivity), and N_c is the effective density of states for the conduction band (2.8•10^{19} for Si). The valence band edge is determined to be 0.05 V vs. SCE using E_{cb} and the Si band gap (1.1 eV).

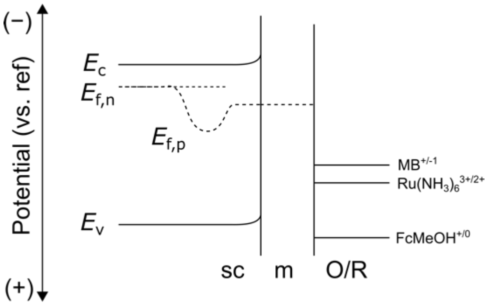

Figure S4: Schematic band diagram showing the approximate band edge positions and redox potentials of the redox species used herein.

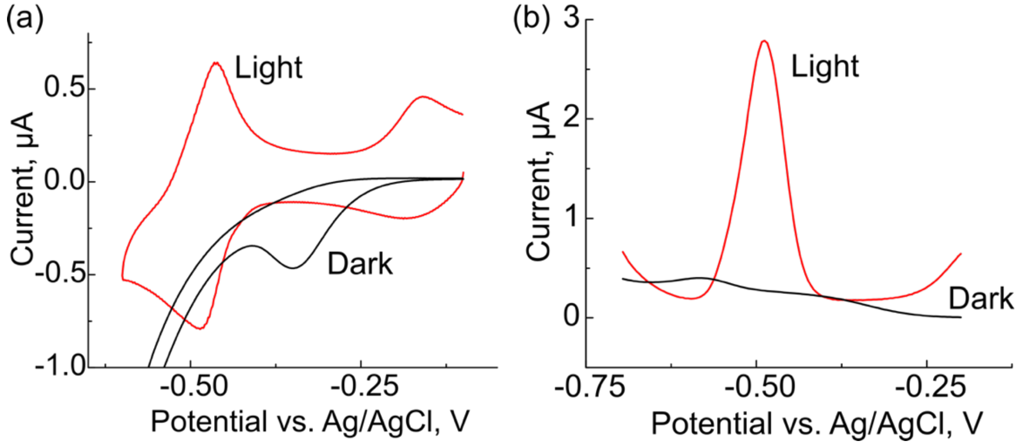

Figure S5: (a) CV and (b) SW voltamograms of 10 µM methylene blue in 1X PBS. Experimental conditions: v = 0.1 V s^{-1}; f = 15 Hz; ΔEp = 25 mV; reference = Ag/AgCl (Sat'd KCl); counter = Pt wire.

Section S3. Calibration of local LED power

The LED light source used for local illumination was calibrated by varying the drive current on the UPLED LED power supply. The light was carefully focused on the surface of a Thorlabs USB power meter (PM16-122) and the intensity was measured at each drive current over the range from 9 to ~100 mA. The light source and power meter were contained in a custom made dark box and the calibrations were performed with the room lights off. Figure S6 shows the results of the experiment. One point calibrations were used before measurements.

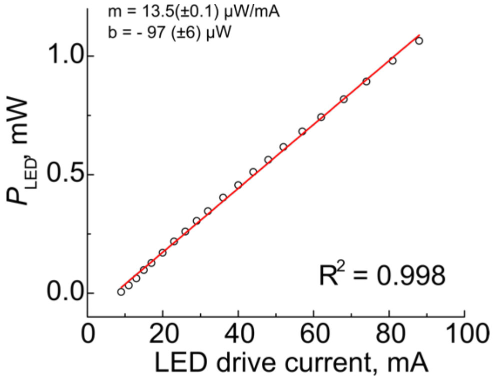

Figure S6: Calibration plot relating LED power to LED drive current.

Section S4. Brief description of square wave voltammetry

SWV is a differential pulsed voltammetric method that enables high sensitivity and low detection limits due to suppression of non-Faradaic background currents.[4] Fig. S7 shows the potential waveform of a SWV experiment, which is a potential step (ΔE_s) with an overlaid square wave, characterized by its amplitude (ΔE_p) and frequency (f). The current is sampled twice per square wave cycle – once on the forward pulse (blue dot) and once on the reverse pulse (red dot). A SW voltammogram is most often presented by plotting $i_{difference}$ (= $i_{forward} - i_{reverse}$) versus the step potential. On anodic scans, the forward pulse is positive and the reverse pulse is negative; on cathodic scans, the forward pulse is negative and the reverse pulse is positive. The frequency of the voltammetric pulse ($f = t_{pulse}^{-1}$) controls the timescale of the experiment and diffusion layer at the electrode surface. The overall voltammetric scan rate in V s^{-1} is a product of the step height (ΔEs) and pulse frequency ($v = \Delta E\text{s}\bullet f$). The pulse amplitude controls the driving force for the reaction relative to the potential step height.

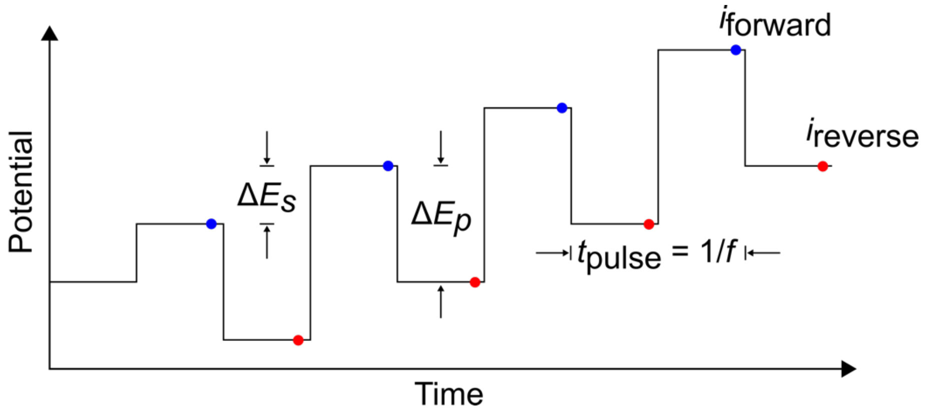

Figure S7: Schematic depiction of the SWV waveform.

Section S5. Additional data for SW parameter study for $Ru(NH_3)_6^{3+}$

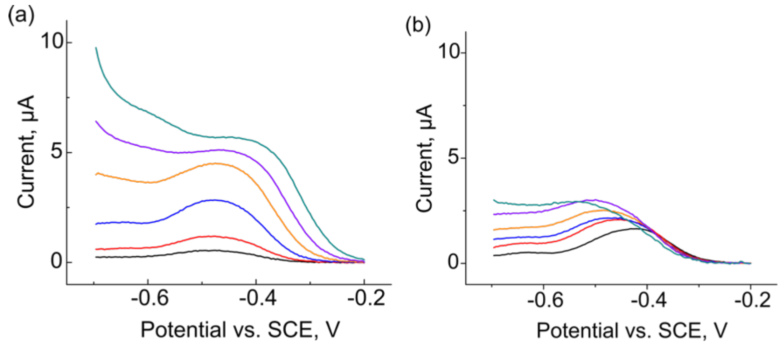

Figure S8: Representative SWVs of $Ru(NH_3)_6^{3+}$ with n-Si/Au NP LAES in the dark collected at (a) f = 15 Hz and ΔEp = 5–100 mV; (b) ΔEp = 25 mV and f from 5–100 Hz.

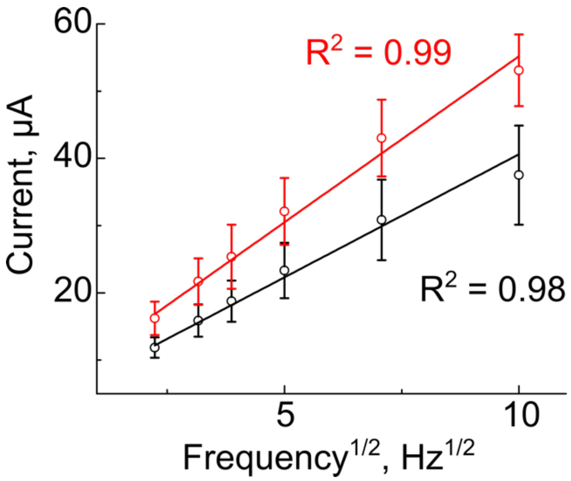

Figure S9: Plot of peak current versus the square root of frequency for $Ru(NH_3)_6^{3+}$. Original data is displayed in Fig. 4f in the main text.

Figs. S10a and S10b shows how $w_{1/2}$ changes with amplitude and frequency, respectively. For nSi/Au NP LAES in the dark (violet circles), $w_{1/2}$ is ~150 mV at low amplitudes and rises to ~200 mV at 100 mV. Upon illumination of the LAES (black circles), $w_{1/2}$ decreases to ~90 mV for amplitudes <25 mV and increases to ~200 mV over the amplitude range from 25 to 100 mV, suggesting at low amplitudes the reaction is nearly reversible. The metallic Si control sample data (red circles) were nearly identical to the illuminated data. Fig. S10b shows that in the dark, nSi/Au NP samples (violet circles) show $w_{1/2}$ is ~150 mV over the entire frequency range, while the illuminated nSi/Au NP samples (black circles) are ~90 mV below 25 Hz and increase to ~150 mV at 100 Hz. The control samples (red circles) show similar trends to the illuminated samples.

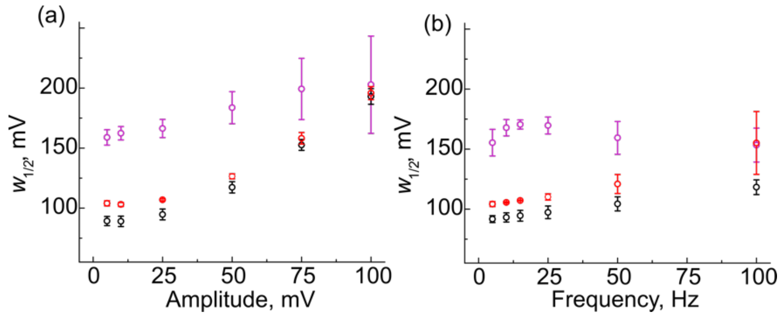

Figure S10: Plot of $w_{1/2}$ versus (a) amplitude and (b) frequency for Ru(NH3)63+ reduction on n-Si/Au NP LAE sensors.

<u>Section S6. Impact of SW amplitude and frequency on the SWV FcMeOH</u>

Representative SW voltammograms for FcMeOH oxidation n-Si/Au NP LAES at varied amplitudes and frequencies are shown in Fig. S11a and S11d, respectively. Also included in Fig. S11a and S11d are SW voltammograms measured using p^+Si/Au NP control samples. These control samples are metallic in nature and are not photoactive. The data are similar to the data presented in the main text for $Ru(NH_3)_6^{3+}$ – the peaks broaden and increase in height as amplitude and frequency increase. The peak broadening with increasing frequency is much less dramatic for FcMeOH than for $Ru(NH_3)_6^{3+}$. The dark current data (Fig. S12) is largely invariant of the SW conditions. The LAES data and p^+Si control data are qualitatively similar, with the control data having a peak center ~0.22 V vs. SCE (i.e., the redox potential of FcMeOH), while the LAES data appears centered at –0.22 V vs. SCE.

Fig S11b and S11e show how $w_{1/2}$ is affected by the square wave amplitude and frequency. Peak width, $w_{1/2}$, is related to the electron transfer kinetics and has a limiting value of ~90 mV for fast 1 e– transfers. At low amplitudes, the $w_{1/2}$ is ~90 mV consistent with a fast one electron redox process. When scanning frequency, the results were more or less constant over the entire frequency range. We did not observe a significant difference for $w_{1/2}$ between the LAES and the p^+Si/Au NP control samples, suggesting

that carrier generation and transport from the Si to Au NPs does not impact the measured current or overall kinetics. This is similar to our previous report, where rate constants were similar for nSi and p$^+$Si control samples.

We next investigated the impact of SW amplitude and frequency on the peak current. As amplitude increases, the peak current increases before leveling off at amplitudes >~50 mV. As frequency increases, the increase in peak current increases linearly with the square root of scan rate ($v = \Delta E_s \bullet f$) consistent with semi-infinite linear diffusion of the redox couple to the electrode surface (Fig. S13). Current is plotted vs. sqrt of frequency, a strong linear correlation is observed ($R^2 > 0.99$ for both samples).

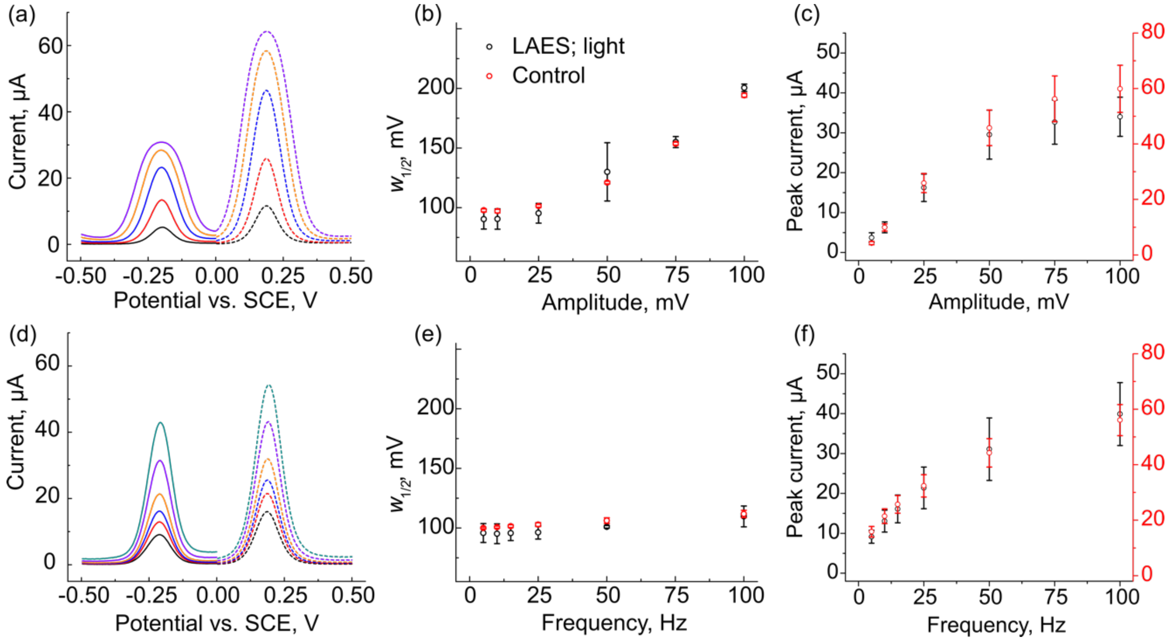

Figure S11: (a) Representative SWVs of 1 mM FcMeOH with illuminated nSi/Au NP LAES (solid lines) and p$^+$Si/Au NP control samples (dashed lines) collected with f = 15 Hz and ΔEp = 5–100 mV; (b) plot of $w_{1/2}$ versus amplitude; (c) plot of i_p versus amplitude; (d) Representative SWVs of 1 mM FcMeOH with illuminated nSi/Au NP LAES (solid lines) and p$^+$Si/Au NP control samples (dashed lines) collected with ΔEp = 25 mV and f from 5–100 Hz; (e) plot of $w_{1/2}$ versus f; (f) plot of i_p versus f. For parts (b), (c), (e), and (f) black circles are the illuminated LAES and the red circles p$^+$Si/Au NP controls.

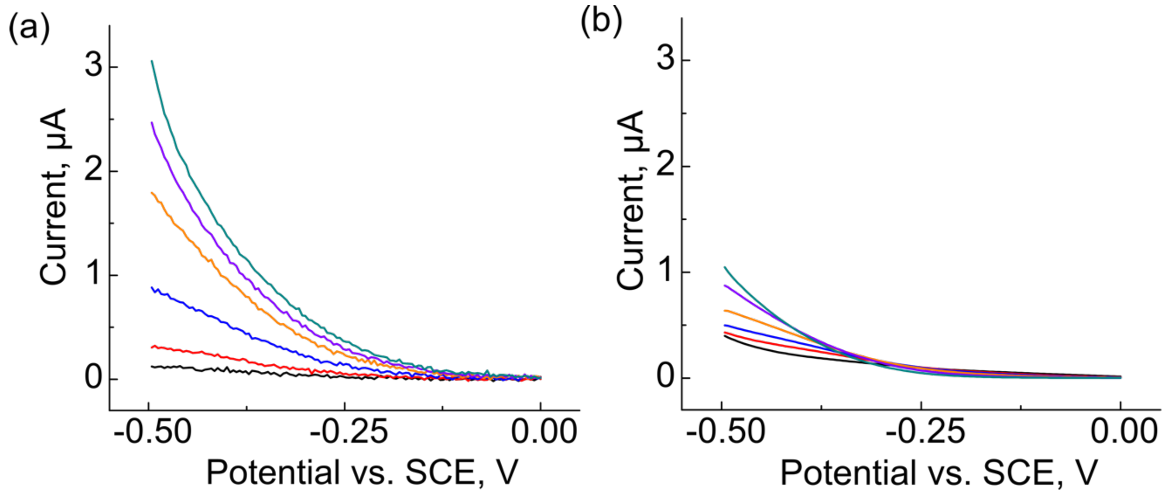

Figure S12: Representative SWVs of FcMeOH with n-Si/Au NP LAES in the dark collected at (a) f = 15 Hz and ΔEp = 5–100 mV; (b) ΔEp = 25 mV and f from 5–100 Hz.

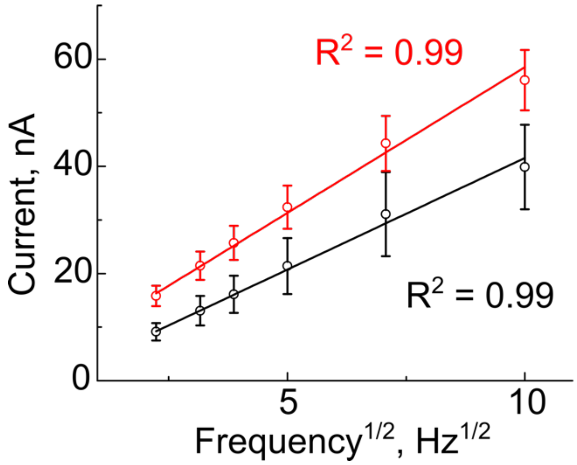

Figure S13: Plot of peak current versus the square root of frequency for FcMeOH. Original data is displayed in Fig. S7.

Section S7. Additional analysis of local illumination SWV measurements

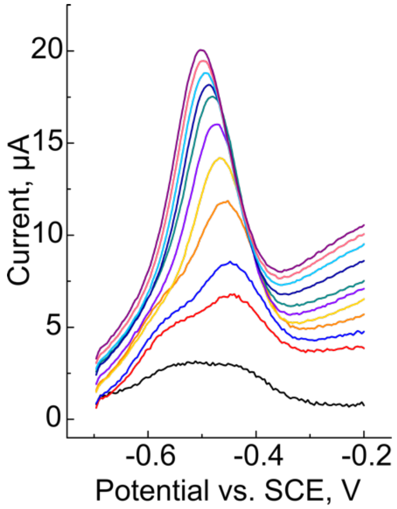

Figure S14: Dark current subtracted SW voltammograms for $Ru(NH_3)_6^{3+}$ reduction at n-Si/Au NP LAES. Data without the dark current subtraction is shown in Fig. 5d in the main text.

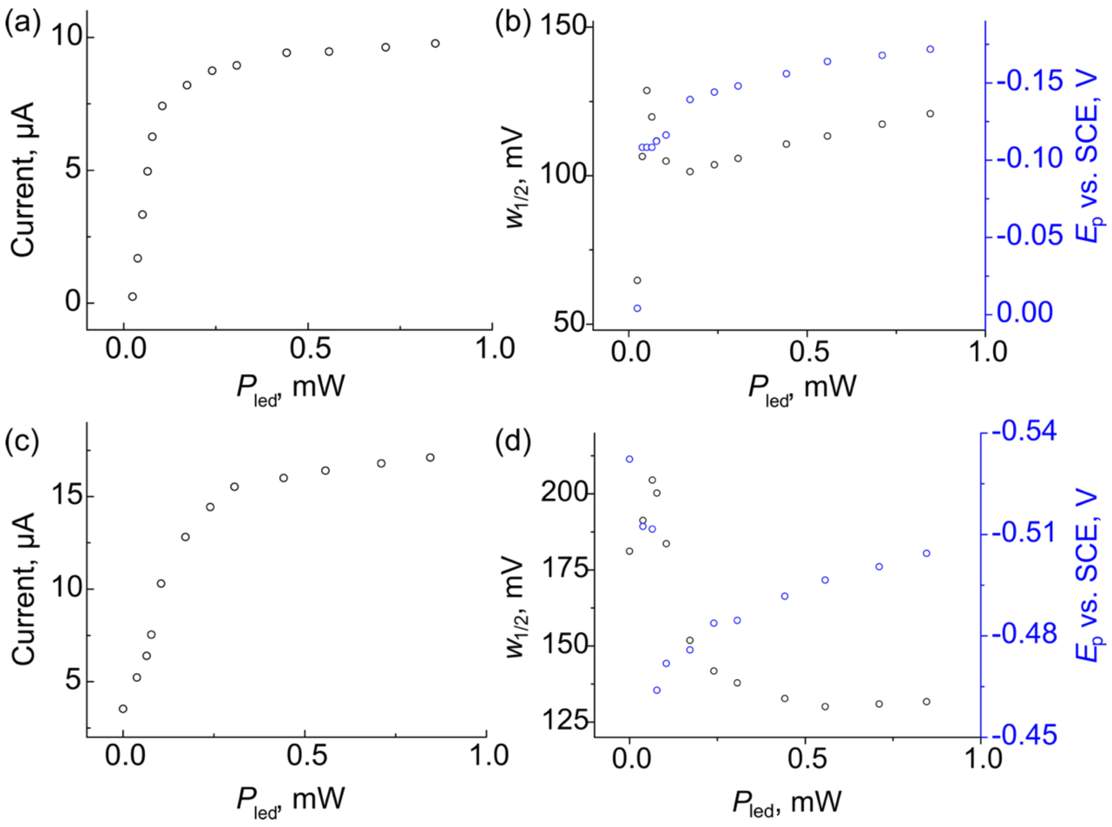

Figure S15: Plots of peak current, (a and c) and $w_{1/2}$ (b and d), versus illumination power for FcMeOH (a and b) and $Ru(NH_3)_6^{3+}$ (c and d).

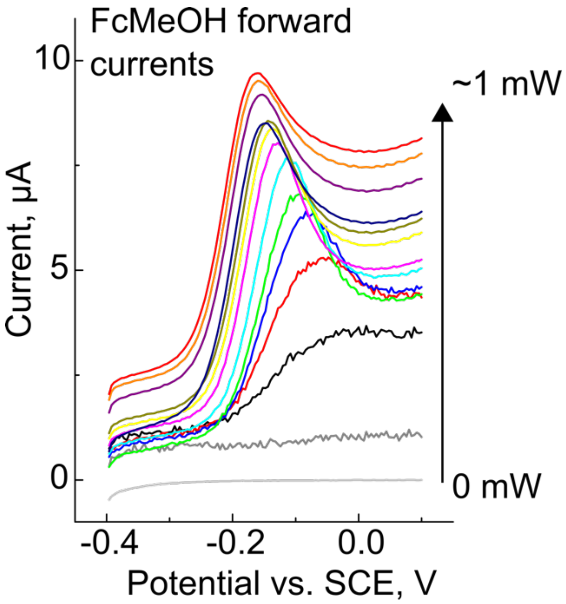

Figure S16: Plots of SWV forward currents for the oxidation of FcMeOH recorded at various illumination intensities between 0 (dark trace) and ~1 mW. Crossover estimate is based on the red trace, where the forward current shows diffusional response ($P \approx 51.5$ µW).

Section S8. References

(1) Terrero Rodríguez, I. M.; Borrill, A. J.; Schaffer, K. J.; Hernandez, J. B.; O'Neil, G. D. Light-Addressable Electrochemical Sensing with Electrodeposited n-Silicon/Gold Nanoparticle Schottky Junctions. *Anal. Chem.* **2020**, *92*, 11444–11452.

(2) Prod'Homme, P.; Maroun, F.; Cortès, R.; Allongue, P. Electrochemical Growth of Ultraflat Au(111) Epitaxial Buffer Layers on H-Si(111). *Appl. Phys. Lett.* **2008**, *93* (17), 21–24.

(3) Hankin, A.; Bedoya-Lora, F. E.; Alexander, J. C.; Regoutz, A.; Kelsall, G. H. Flat Band Potential Determination: Avoiding the Pitfalls. *J. Mater. Chem. A* **2019**, *7* (45), 26162–26176.

(4) Osteryoung, J.; Osteryoung, R. A. Sqaure Wave Voltammetry. *Anal. Chem.* **1985**, *57* (1), 101A-110A.
